# Machine learning models of Alzheimer's disease spectrum using blood tests

**DOI:** 10.1002/dad2.70228

**Published:** 2025-12-09

**Authors:** Nicola Walter Falasca, Antonio Ferretti, Alberto Granzotto, Stefano L. Sensi, Raffaella Franciotti, for the Alzheimer's Disease Neuroimaging Initiative (ADNI)

**Affiliations:** ^1^ Department of Neuroscience, Imaging, and Clinical Sciences G. d'Annunzio University of Chieti‐Pescara Chieti Italy; ^2^ UdA‐TechLab Research Center University "G. d'Annunzio" of Chieti‐Pescara Chieti Italy; ^3^ Institute for Advanced Biomedical Technologies G. d'Annunzio University of Chieti‐Pescara Chieti Italy; ^4^ Center for Advanced Studies and Technology – CAST G. d'Annunzio University of Chieti‐Pescara Chieti Italy; ^5^ Institute of Neurology SS Annunziata University Hospital, Chieti, University of Chieti‐Pescara Chieti Italy

**Keywords:** apolipoprotein E (*APOE*) ε4 allele, cholesterol‐related analytes, circulating peripheral biomarkers, fatty acids, glycolysis‐related metabolites, mild cognitive impairment (MCI), supervised support vector machine (SVM) algorithm

## Abstract

**INTRODUCTION:**

The diagnosis of Alzheimer's disease (AD) traditionally relies on cerebrospinal fluid and plasma levels of amyloid beta and phosphorylated tau. Although informative, these biomarkers represent a narrow, hypothesis‐driven approach to intercept the disease.

**METHODS:**

Data‐driven analysis was applied on demographic data, apolipoprotein E (*APOE*) ε4 allele, and 82 biomarkers obtained from blood tests of healthy controls (HC), mild cognitive impairment that remained stable within 36 months following blood collection (sMCI), and patients with AD.

**RESULTS:**

Statistical analyses revealed differences among groups in many cholesterol‐related analytes. *APOE* ε4 and analytes such as amino acids, lipoproteins, and fatty acids emerged as the most influential features in machine learning (ML) classification algorithms. Glycolysis‐related metabolites and amino and fatty acids were predictive for distinguishing sMCI and AD from HC.

**DISCUSSION:**

These findings support the hypothesis that systemic alterations also occur during the preclinical stages of dementia, which can be detected by ML models on blood biomarkers.

**Highlights:**

- Machine learning on blood tests detects preclinical cognitive decline.
- Glycolysis metabolites are predictive for distinguishing stable MCI and AD from HC.
- Amino acids, lipoproteins, and fatty acids are the most predictive features.
- Inflammatory and metabolic biomarkers represent a biosignature of cognitive health.

## 1 BACKGROUND

Imaging and molecular biomarkers, obtained from cerebrospinal fluid (CSF) or blood samples, are entering clinical practice to support physicians in the diagnosis of Alzheimer's disease (AD).^1^, ^2^ Assays aimed at evaluating amyloid beta (Aβ) and phosphorylated tau (p‐tau) levels with positron emission tomography (PET)–based tracers or from biological fluids are valuable for the early diagnosis of AD, for monitoring, and for distinguishing the condition from other forms of dementia.^3^ In addition, there is great interest in the prognostic utility of such tools, with p‐tau–based models holding great promise as predictors of the future development of AD dementia in individuals with early signs of cognitive decline.^3^ However, the use of imaging or peripheral biomarkers remains challenging and often limited to highly specialized clinical facilities. Compared to amyloid PET and CSF testing, blood biomarkers have significant practical advantages that may democratize the use of testing. These include the acceptability of blood draws by patients, accessibility of equipment and staff, and lower costs.^4^

On the AD continuum, mild cognitive impairment (MCI) is identified as a transition stage between healthy aging and dementia.^5^ Considered as the gateway to AD dementia, the MCI phase has drawn substantial attention for studies aimed at identifying prognostic biomarkers, with critical implications for diagnosis and patient management. Among the increasing number of genetic risk factors for AD, the apolipoprotein E (*APOE*) gene, which is associated with various cellular functions related to immune responses, endocytosis, and cholesterol metabolism,^6^ remains the strongest and most prevalent and recently has been identified as an across‐disease trigger of neurodegeneration.^7^ Heterozygosity and homozygosity for the ε4 allele of the *APOE* gene increase AD risk by 3‐ and 12‐fold, respectively.^8^ *APOE* ε2 confers protection, whereas *APOE* ε3 is considered neutral.^9^

Emerging evidence suggests that AD's etiology transcends the traditional linear model of Aβ dysmetabolism, leading to p‐tau accumulation and neuronal death.^10^, ^11^ More likely, multiple central and peripheral biological pathways—including cerebrovascular pathology, inflammation, vascular and endothelial injury, glucose metabolism, insulin signaling, and lipid processing— synergistically contribute to the AD‐related cognitive decline.^12^, ^13^, ^14^, ^15^ However, the intricate, nonlinear interactions among these pathways pose challenges in fully understanding the molecular changes and their roles in disease progression.^16^

In this context, computer‐based statistical methods known as machine learning (ML) algorithms offer significant advantages for analyzing large datasets of biomarkers, looking for recurring patterns. The ability of these algorithms to handle high‐dimensional data and effectively classify complex, nonlinear relationships make these tools particularly well‐suited for predicting outcomes in large cohorts. However, current ML applications in publicly available datasets, like the Alzheimer's Disease Neuroimaging Initiative (ADNI) cohort, face critical gaps. First, most studies prioritize novel, specialized biomarkers (e.g., p‐tau217, neurofilament light chain [NfL], and glial fibrillary acidic protein [GFAP]) over routinely available clinical blood analytes despite their clinical practicality. Second, there is limited exploration of how AD‐risk genotypes, like *APOE* status, modulate routine biomarker patterns across disease stages. Third, existing models often lack reproducibility due to proper biobanking, appropriate blood handling, and preprocessing.^17^, ^18^

This observational cross‐sectional study aims at identifying possible differences in blood analyte values among healthy controls (HC), MCI individuals which remained stable within 36 months after blood collection (sMCI), and AD patients, as well the best combination of routinely performed and research‐grade circulating biomarkers helpful for classifying each group by employing supervised ML algorithms. The study, leveraging on the wealth of standardized information obtained from the ADNI dataset (https://adni.loni.usc.edu) aims to identify clinically accessible and cost‐effective blood analytes that can aid in predicting the course of AD.

## 2 METHODS

### 2.1 Participants

Demographic information (age, sex, education, ethnicity, and race), *APOE* ε4 genotype, Aβ, p‐tau, and 82 analytes from blood tests were obtained from the ADNI database for HC, sMCI, and AD patients. All the data used in this study are available from the ADNI database. HC, sMCI, and AD subjects were then selected for the study. HC and sMCI patients were included only if they remained stable within 36 months following blood collection. AD patients were included if they had not changed their diagnosis in the follow‐up visits.

**RESEARCH IN CONTEXT**

1. **Systematic review**: The diagnosis of mild cognitive impairment (MCI) and dementia due to Alzheimer's disease (AD) relies on a hypothesis‐driven approach quantifying tau and amyloid in cerebrospinal fluid, plasma, and imaging scans. However, the use of these biomarkers remains challenging and often limited to highly specialized clinical facilities.
2. **Interpretation**: The current data‐driven study indicated that machine learning algorithms applied to blood test analytes can effectively distinguish individuals with MCI and AD from healthy controls. The findings showed that immune, inflammatory, and metabolic biomarkers collectively represent a biological signature of cognitive health. This approach can potentially streamline the diagnostic process and make early detection more feasible in diverse clinical settings.
3. **Future directions**: Future research should integrate non‐invasive circulating biomarkers with amyloid beta deposition, tau accumulation, and neurofilament light chain levels to enhance our understanding of the relationship between peripheral alterations and AD pathology, potentially leading to more accurate and early diagnoses.

The ADNI dataset was launched in 2003 as a public–private partnership led by Principal Investigator Michael W. Weiner, MD. The clinical coordination center of ADNI established a network of clinical sites. It developed the clinical protocol and informed consent, which is distributed to the sites for local institutional review board ethical approval. One such institution is the Office for the Protection of Research Subjects at the University of Southern California. Participants provided written informed consent for the study. More details can be found at adni.loni.usc.edu. The primary goal of the ADNI has been to test whether serial magnetic resonance imaging (MRI), PET, other biological markers, and clinical and neuropsychological assessment can be combined to measure the progression of MCI and early AD.

### 2.2 Peripheral biomarkers

Peripheral biomarkers included 82 analytes belonging to 12 major metabolic groups: amino acids, apolipoproteins, cholesterol, fatty acids, fluid balance, glycolysis metabolites, ketone bodies, lipoprotein, other lipids, phospholipids, total lipids, and triglycerides. All variables are listed in Table 1. In a subset of the study cohort, Aβ42 and p‐tau181 values from CSF were also included.

**TABLE 1:** List of all analytes included in the prediction models.

Metabolite group	Analyte	Assay availability	Origin
Amino acids	ALA, Alanine (mmol/L)	Research‐grade metabolomics	LC‐MS
	GLN, Glutamine (mmol/L)	Research‐grade metabolomics	LC‐MS
	GLY, Glycine (mmol/L)	Research‐grade metabolomics	LC‐MS
	HIS, Histidine (mmol/L)	Research‐grade metabolomics	LC‐MS
	ILE, Isoleucine (mmol/L)	Research‐grade metabolomics	LC‐MS
	LEU, Leucine (mmol/L)	Research‐grade metabolomics	LC‐MS
	TOTAL BCAA, Total concentration of branched‐chain amino acids (leucine + isoleucine + valine) [mmol/l]	Research‐grade metabolomics	LC‐MS
	VAL, Valine (mmol/L)	Research‐grade metabolomics	LC‐MS
	PHE, Phenylalanine (mmol/L)	Research‐grade metabolomics	LC‐MS
	TYR, Tyrosine (mmol/L)	Research‐grade metabolomics	LC‐MS
Apolipoproteins	APOA1, Apolipoprotein A1 (g/L)	Research‐grade metabolomics	LC‐MS
	APOB, Apolipoprotein B (g/L)	Research‐grade metabolomics	LC‐MS
	APOB_BY_APOA1, Ratio of apolipoprotein B to apolipoprotein A1	Research‐grade metabolomics	LC‐MS
Cholesterol	LDL C, low‐density lipoprotein (LDL) cholesterol (mmol/L)	Routine clinical test	Standard clinical chemistry
	LDL CE, Cholesteryl esters LDL (mmol/L)	Research‐grade metabolomics	LC‐MS
	LDL FC, Free cholesterol in LDL (mmol/L)	Research‐grade metabolomics	LC‐MS
	NON HDL C, Total cholesterol minus HDL‐C (mmol/L)	Research‐grade metabolomics	LC‐MS
	REMNANT C, Remnant cholesterol (non‐HDL, non‐LDL ‐cholesterol) (mmol/L)	Research‐grade metabolomics	LC‐MS
	TOTAL C, Total cholesterol (mmol/L)	Routine clinical test	Standard clinical chemistry
	TOTAL CE, Total esterified cholesterol (mmol/L)	Research‐grade metabolomics	LC‐MS
	TOTAL FC, Total free cholesterol (mmol/L)	Research‐grade metabolomics	LC‐MS
	VLDL C, Very Low‐Density Lipoprotein (VLDL) cholesterol (mmol/L)	Research‐grade metabolomics	LC‐MS
	VLDL CE, Cholesteryl esters in VLDL (mmol/L)	Research‐grade metabolomics	LC‐MS
	VLDL FC, Free cholesterol in VLDL (mmol/L)	Research‐grade metabolomics	LC‐MS
	HDL CE, Cholesteryl esters in high‐density lipoprotein (HDL) (mmol/L)	Research‐grade metabolomics	LC‐MS
	HDL FC, Free cholesterol in HDL (mmol/L)	Research‐grade metabolomics	LC‐MS
	CLINICAL LDL C, Clinical LDL cholesterol (mmol/L)	Routine clinical test	Standard clinical chemistry
Fatty acids	LA, Linoleic acid (mmol/L)	Research‐grade metabolomics	LC‐MS
	LA PCT, Ratio of linoleic acid to total fatty acids (%)	Research‐grade metabolomics	LC‐MS
	MUFA, Monounsaturated fatty acids (mmol/L)	Research‐grade metabolomics	LC‐MS
	MUFA PCT, Ratio of monounsaturated fatty acids to total fatty acids (%)	Research‐grade metabolomics	LC‐MS
	OMEGA 3 (mmol/L)	Research‐grade metabolomics	LC‐MS
	OMEGA 3 PCT, Ratio of omega‐3 fatty acids to total fatty acids (%)	Research‐grade metabolomics	LC‐MS
	OMEGA 6 (mmol/L)	Research‐grade metabolomics	LC‐MS
	OMEGA 6 BY OMEGA 3, Ratio of omega‐6 fatty acids to omega‐3 fatty acids	Research‐grade metabolomics	LC‐MS
	OMEGA 6 PCT, Ratio of omega‐6 fatty acids to total fatty acids (%)	Research‐grade metabolomics	LC‐MS
	PUFA, Polyunsaturated fatty acids (mmol/L)	Research‐grade metabolomics	LC‐MS
	PUFA BY MUFA, Ratio of polyunsaturated fatty acids to monounsaturated fatty acids	Research‐grade metabolomics	LC‐MS
	PUFA PCT, Ratio of polyunsaturated fatty acids to total fatty acids (%)	Research‐grade metabolomics	LC‐MS
	SFA, Saturated fatty acids (mmol/L)	Research‐grade metabolomics	LC‐MS
	SFA PCT, Ratio of saturated fatty acids to total fatty acids (%)	Research‐grade metabolomics	LC‐MS
	DHA, Docosahexaenoic acid (mmol/L)	Research‐grade metabolomics	LC‐MS
	DHA PCT, Ratio of docosahexaenoic acid to total fatty acids (%)	Research‐grade metabolomics	LC‐MS
	TOTAL FA, Total fatty acids (mmol/L)	Research‐grade metabolomics	LC‐MS
	UNSATURATION, Degree of unsaturation	Research‐grade metabolomics	LC‐MS
Fluid Balance	ALBUMIN, (g/L)	Routine clinical test	Standard clinical chemistry
	CREATININE, (µmol/L)	Routine clinical test	Standard clinical chemistry
Glycolysis‐related metabolites	CITRATE, (mmol/L)	Research‐grade metabolomics	LC‐MS
	GLUCOSE, (mmol/L)	Routine clinical test	Standard clinical chemistry
	GLYCEROL, (mmol/L)	Research‐grade metabolomics	LC‐MS
	LACTATE, (mmol/L)	Research‐grade metabolomics	LC‐MS
	PYRUVATE, (mmol/L)	Research‐grade metabolomics	LC‐MS
	GLYCA, Glycoprotein acetyls (mmol/L)	Research‐grade metabolomics	LC‐MS
Ketone bodies	ACETATE, (mmol/L)	Research‐grade metabolomics	LC‐MS
	ACETOACETATE, (mmol/L)	Research‐grade metabolomics	LC‐MS
	ACETONE, (mmol/L)	Research‐grade metabolomics	LC‐MS
	BOHBUTYRATE, 3‐Hydroxybutyrate (mmol/L)	Research‐grade metabolomics	LC‐MS
Lipoprotein	HDL‐P, Concentration of HDL particles (mmol/L)	Research‐grade metabolomics	LC‐MS
	HDL‐C, Cholesterol in very large HDL (mmol/L)	Routine clinical test	Standard clinical chemistry
	HDL SIZE, Average diameter for HDL particles (nm)	Research‐grade metabolomics	LC‐MS
	LDL‐P, Concentration of LDL particles (mmol/L)	Research‐grade metabolomics	LC‐MS
	LDL SIZE, Average diameter for LDL particles (nm)	Research‐grade metabolomics	LC‐MS
	VLDL‐P, Concentration of VLDL particles (mmol/L)	Research‐grade metabolomics	LC‐MS
	VLDL SIZE, Average diameter for VLDL particles (nm)	Research‐grade metabolomics	LC‐MS
	TOTAL P, Total concentration of lipoprotein particles (mmol/L)	Research‐grade metabolomics	LC‐MS
Other lipids	CHOLINES, Total cholines (mmol/L)	Research‐grade metabolomics	LC‐MS
	PHOSPHATIDYLC, Phosphatidylcholines (mmol/L)	Research‐grade metabolomics	LC‐MS
	PHOSPHOGLYC, Phosphoglycerides (mmol/L)	Research‐grade metabolomics	LC‐MS
	SPHINGOMYELINS, (mmol/L)	Research‐grade metabolomics	LC‐MS
	TG BY PG, Ratio of triglycerides to phosphoglycerides	Research‐grade metabolomics	LC‐MS
Phospholipids	HDL‐PL, Phospholipids in HDL (mmol/L)	Research‐grade metabolomics	LC‐MS
	VLDL‐PL, Phospholipids in VLDL (mmol/L)	Research‐grade metabolomics	LC‐MS
	TOTAL PL, Total phospholipids in lipoprotein particles (mmol/L)	Research‐grade metabolomics	LC‐MS
	LDL‐PL, Phospholipids in LDL (mmol/L)	Research‐grade metabolomics	LC‐MS
Total lipids	HDL L, Total lipids in HDL (mmol/L)	Research‐grade metabolomics	LC‐MS
	LDL L, Total lipids in LDL (mmol/L)	Research‐grade metabolomics	LC‐MS
	TOTAL L, Total lipids in lipoprotein particles (mmol/L)	Research‐grade metabolomics	LC‐MS
	VLDL L, Total lipids in VLDL (mmol/L)	Research‐grade metabolomics	LC‐MS
Triglycerides	HDL TG, Triglycerides in HDL (mmol/L)	Research‐grade metabolomics	LC‐MS
	LDL TG, Triglycerides in LDL (mmol/L)	Research‐grade metabolomics	LC‐MS
	TOTAL TG, Total triglycerides (mmol/L)	Routine clinical test	Standard clinical chemistry
	VLDL TG, Triglycerides in VLDL (mmol/L)	Research‐grade metabolomics	LC‐MS

### 2.3 Statistical analysis

Data are reported as mean ± standard deviation (SD) or percentage for continuous and dichotomous variables. Age, education, Aβ42, and p‐tau181 were compared among the three groups (HC, sMCI, and AD) using univariate analysis of variance (ANOVA). Receiver‐operating characteristic (ROC) analyses were performed on Aβ and p‐tau values to estimate the diagnostic accuracy of these biomarkers in the selected sample.

Multivariate analysis of covariance (MANCOVA) was performed to compare analytes among groups. Thus, analytes were used as multiple dependent variables; group (HC, sMCI, and AD) as an independent variable; and age, sex, education, ethnicity, and race as covariates. The assumption of homogeneity of variance across the groups was verified by Levene's test. The Bonferroni test was applied for the post hoc comparisons. Nonparametric statistics were applied for the comparison of dichotomous variables (sex, *APOE* ε4 status, ethnicity, and race) among the three groups. The Kruskal–Wallis test was applied for the post hoc analyses.

The significant analytes obtained from MANOVA and with the largest effect size among analytes in the same metabolite group were correlated with *APOE* ε4 status (absence or presence of at least one ε4 allele), correcting for age, sex, education, ethnicity, and race. These analytes were also correlated with education, correcting for age, sex, ethnicity, and race. For all statistical comparisons, the level of significance was set at 0.05.

### 2.4 ML algorithms

Optimized support vector machine (SVM) algorithm, implemented in Matlab 2023, was applied to the dataset, including all 82 analytes, age, sex, education, and *APOE* ε4 status. An ML model was estimated for each of the following binary classifications: sMCI versus HC, AD versus HC, and AD versus sMCI. As the first step, the algorithm splits the entire dataset into train, validation, and test datasets randomly. Data were used for the training (70% of the subjects) and for the validation phase (15% of subjects). SVM algorithms were used to train and validate the models. Specifically, optimized SVM was used to find the best hyperparameter values that improve the performance of the model. We used a linear Kernel function and 30 iterations in the Bayesian optimization process. This Bayesian approach is an advanced hyperparameter tuning technique which allows for evaluating the object function, reducing the search process within the parameter space.^19^ This step is critical because ML models need to set hyperparameters to best tune the algorithm for data testing. After building the model, the testing phase was applied to the remaining 15% of the dataset.

To evaluate the performance of each ML algorithm, accuracy (ACC), positive predictive value (PPV), negative predictive value (NPV), sensitivity, specificity, and area under the ROC curve (AUC) were calculated for each model in the test datasets.

### 2.5 Predictive features

The classification algorithm that obtained the best accuracy after the tuning procedure was selected for further analysis using fewer variables. The ReliefF test was used as a feature‐ranking algorithm to calculate a feature score for each variable. ReliefF is an evaluation filter algorithm capable of detecting feature dependencies using the concept of nearest neighbors, and selects top‐scoring features (feature selection procedure). The output score ranges from −1 to 1, with more positive scores indicating more predictive features. We used a forward selection process that retains an additional variable as a predictive feature only if it improves the model's performance, increasing AUC by at least 1%, compared to that of the previous model obtained so far.

The SHAP (SHapley Additive exPlanations) algorithm^20^ was applied to provide a consistent and objective explanation of how each feature impacts the model's prediction. SHAP assigns an importance value to each feature, indicating how much each feature predicts the target. The magnitude of the SHAP value is a measure of how strong the effect is. Features with positive SHAP values improve the prediction, whereas those with negative values worsen the prediction.

## 3 RESULTS

### 3.1 Statistical results

**FIGURE 1:**
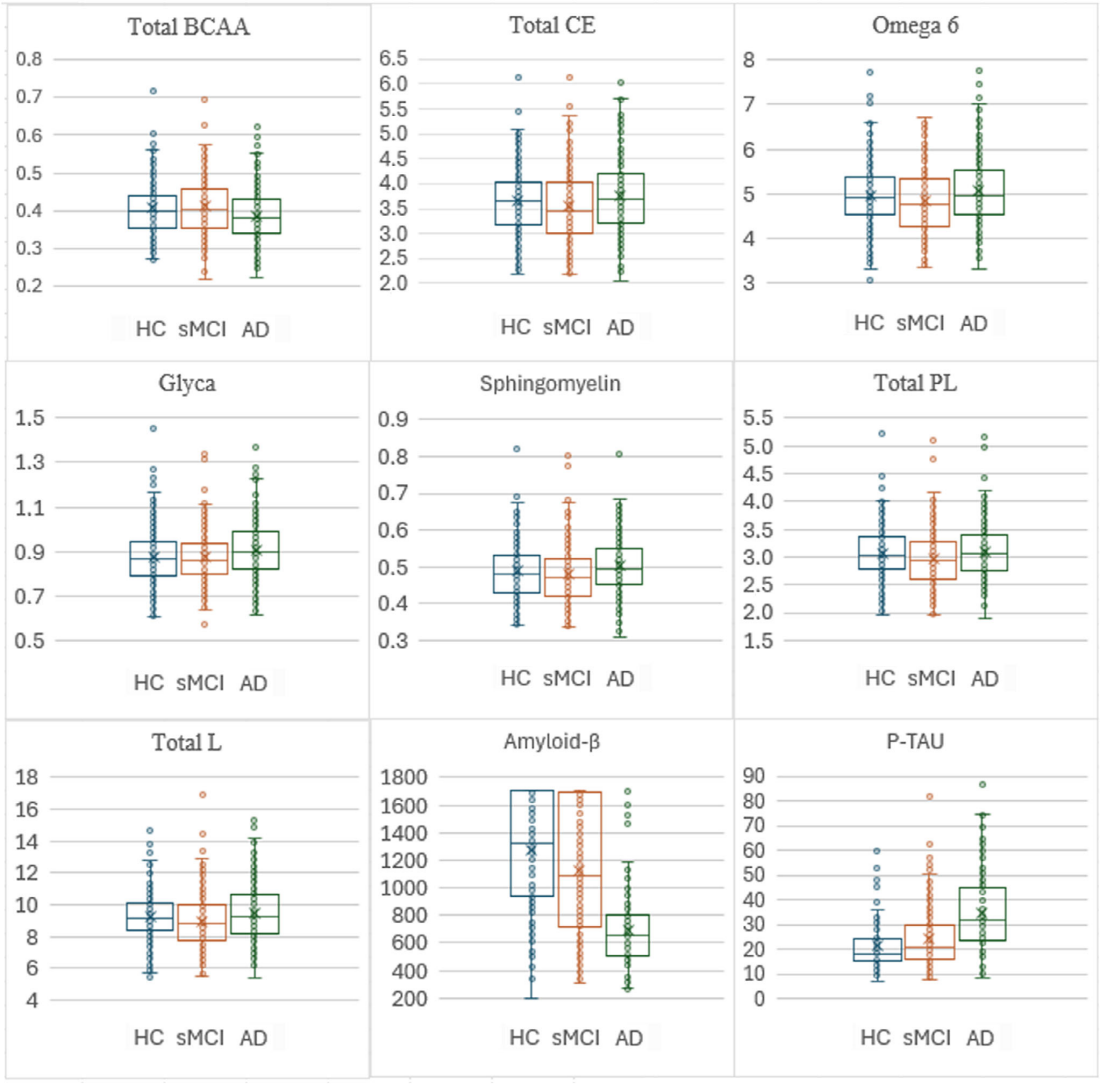
The boxplot distribution of the significant analytes showing the best effect size for each metabolite group. AD, Alzheimer's disease; glyca, glycoprotein acetyls; HC, healthy control; P‐TAU, phosphorylated tau; sMCI, stable mild cognitive impairment; TOTAL BCAA, total concentration of branched‐chain amino acids (leucine + isoleucine + valine); TOTAL CE, total esterified cholesterol; TOTAL L, total lipids in lipoprotein particles; TOTAL PL, total phospholipids in lipoprotein particles.

Age, sex, and *APOE* ε4 status were statistically different among groups. sMCI subjects were younger when compared to AD patients (*p* = 0.02), female prevalence was higher in HC than in sMCI (*p* = 0.001) and AD (*p* = 0.01), and *APOE* ε4 prevalence was lower in HC than in sMCI and AD, and higher in AD than in sMCI (all *p* values = 0.001). Statistical analyses on analytes using age, sex, education, ethnicity, and race as covariates showed significant differences among cohorts on seven metabolite groups (i.e., amino acids, cholesterol, fatty acids, glycolysis‐related metabolites, other lipids, phospholipids, and total lipids). Post hoc analyses showed that all analytes were significantly different between the AD, HC, and sMCI groups. Total concentration of branched‐chain amino acids (BCAAs, leucine + isoleucine + valine), total esterified cholesterol, omega 6, glycoprotein acetyls (glyca), sphingomyelin, total phospholipids in lipoprotein particles, and total lipids in lipoprotein particles were the analytes with the best effect size for each metabolite group. The boxplot distribution of these analytes is shown in Figure 1. No significant difference was found between sMCI and HC. Participants’ characteristics and statistical results are shown in Table 2. No significant partial correlation was found between the analytes and *APOE* ε4. Instead, significant partial correlation was found between total esterified cholesterol and *APOE* ε4 when correcting for sex (*ρ* = 0.076, *p* = 0.038). Significant partial correlations were also found between education and BCAA (*ρ* = –0.073, *p* = 0.045) in cholesterol group, omega 6 (*ρ* = –0.159, *p* = 0.013·10^−3^) in fatty acids group, glyca (*ρ* = –0.151, *p* = 0.034·10^−3^) in glycolysis‐related metabolites, and total lipids in lipoprotein particles (*ρ* = −0.110, *p* = 0.003).

**TABLE 2:** Participants’ characteristics (mean ± SD) and statistical results on demographic variables and analytes corrected for age, sex, education, ethnicity, and race.

					Post hoc		
Characteristic	HC (*n* = 250)	sMCI (*n* = 250)	AD (*n* = 250)	Main effect	HC vs sMCI	HC vs AD	sMCI vs AD
**Demographic**							
Age^a^	73.4 ± 5.7	72.9 ± 7.3	74.5 ± 7.2	0.02	0.9	0.2	**0.01**
Sex, male %^b^	46.4	63.2	59.2	0.01·10^−1^	**0.01·10^−1^**	**0.01**	1.0
Education, y^a^	16.4 ± 2.7	16.0 ± 2.7	15.9 ± 2.7	0.04	0.16	0.05	1.0
Ethnicity^b^				0.25			
Hispanic	11 (4%)	7 (3%)	7 (3%)				
Non‐Hispanic	237 (95%)	243 (97%)	243 (97%)				
Unknown	2 (1%)	0	0				
Race^b^				0.30			
Black	12 (5%)	5 (2%)	5 (2%)				
American Indian	1 (0%)	1 (0%)	0				
Asian	3 (1%)	5 (2%)	4 (2%)				
White	232 (93%)	237 (95%)	240 (96%)				
More than one	2 (1%)	2 (1%)	1 (0%)				
**Genetic** ^b^							
*APOE* ε4 (%)	23.2	44.0	63.2	0.01·10^−1^	**0.01·10^−1^**	**0.01·10^−1^**	**0.01·10^−1^**
**Metabolite group** ^c^							
Analyte							
Amino acids							
LEU	0.12 ± 0.04	0.12 ± 0.03	0.11 ± 0.02	7.87·10^−12^	1.00	**7.06·10^−4^**	**7.79·10^−3^**
**TOTAL BCAA**	0.41 ± 0.08	0.41 ± 0.08	0.39 ± 0.07	3.06·10^−13^	1.00	**1.02·10^−3^**	**6.05·10^−3^**
VAL	0.23 ± 0.04	0.24 ± 0.04	0.22 ± 0.04	7.11·10^−11^	1.00	**6.85·10^−4^**	**3.71·10^−3^**
Cholesterol							
Clinical LDL C	2.78 **± **0.66	2.73 ± 0.73	2.91 ± 0.79	1.16·10^−15^	1.00	**1.97·10^−2^**	**1.13·10^−2^**
LDL C	1.94 ± 0.41	1.91 ± 0.45	2.02 ± 0.50	1.04·10^−15^	1.00	**1.77·10^−2^**	**1.31·10^−2^**
LDL CE	1.42 ± 0.30	1.40 ± 0.33	1.48 ± 0.37	1.60·10^−15^	1.00	**2.13·10^−2^**	**1.49·10^−2^**
LDL FC	0.52 ± 0.11	0.51 ± 0.13	0.54 ± 0.13	3.10·10^−15^	1.00	**1.46·10^−2^**	**1.28·10^−2^**
NON HDL C	3.41 ± 0.73	3.33 ± 0.82	3.54 ± 0.91	4.37·10^−17^	1.00	**3.72·10^−2^**	**7.40·10^−3^**
TOTAL C	4.96 ± 0.88	4.81 ± 0.98	5.10 ± 1.01	4.42·10^−29^	1.00	**1.27·10^−2^**	**9.36·10^−4^**
**TOTAL CE**	3.66 ± 0.65	3.54 ± 0.72	3.75 ± 0.73	4.38·10^−30^	1.00	**1.11·10^−2^**	**1.05·10^−3^**
TOTAL FC	1.31 ± 0.24	1.27 ± 0.27	1.35 ± 0.29	4.78·10^−25^	1.00	**2.33·10^−2^**	**1.05·10^−3^**
Fatty acids							
LA	3.99 ± 0.73	3.86 ± 0.77	4.13 ± 0.82	2.14·10^−21^	5.96·10^−1^	**3.05·10^−2^**	**3.16·10^−4^**
OMEGA 6	4.94 ± 0.73	4.82 ± 0.75	5.06 ± 0.79	2.21·10^−29^	9.32·10^−1^	**2.48·10^−2^**	**7.10·10^−4^**
Glycolysis							
Glyca	0.88 ± 0.13	0.87 ± 0.12	0.91 ± 0.13	1.22·10^−13^	1.00	**6.02·10^−3^**	**1.59·10^−2^**
Other lipids							
Sphingomyelin	0.49 ± 0.08	0.48 ± 0.08	0.50 ± 0.08	5.79·10^−34^	1.00	**9.34·10^−4^**	**5.56·10^−4^**
Phospholipids							
LDL PL	0.67 ± 0.13	0.66 ± 0.14	0.70 ± 0.16	4.59·10^−15^	1.00	**1.79·10^−2^**	**1.41·10^−2^**
**TOTAL PL**	3.07 ± 0.47	2.95 ± 0.50	3.10 ± 0.51	1.98·10^−40^	6.30·10^−1^	8.21×10^−2^	**1.50×10^−3^**
Total lipids							
LDL L	2.76 ± 0.55	2.73 ± 0.61	2.87 ± 0.68	8.48·10^−30^	1	**1.87·10^−2^**	**1.25·10^−2^**
**TOTAL L**	9.23 ± 1.52	8.92 ± 1.65	9.43 ± 1.74	4.75×10^−6^	6.50·10^−1^	**4.68·10^−2^**	**7.17·10^−4^**
	HC (n = 122)	sMCI (198)	AD (n = 139)	Main Effect	HC vs. sMCI	HC vs. AD	sMCI vs. AD
Aβ42^a^ (ng/L)	1267.9 ± 37.6	1119.8 ± 32.6	701.1 ± 25.1	5.71×10^−28^	**0.05×10^−1^**	**7.29×10^−26^**	**9.91×10^−19^**
p‐tau181^a^ (ng/L)	21.3 ± 0.9	24.5 ± 0.8	35.0 ± 1.3	1.94×10^−18^	0.08	**8.87×10^−17^**	**6.95×10^−13^**

*Notes*: For glyca in the glycolysis pη^2 ^= 0.10, and for sphingomyelin in the other lipids metabolite group pη^2 ^= 0.21.

For each metabolite group analytes with the greatest pη^2^ values are shown in bold. Significant *p*‐values in the post hoc comparisons are shown in bold.

Abbreviations: Aβ42, amyloid‐β42; AD, Alzheimer's disease; APOE ε4, Apolipoprotein E ε4 genotype; AUC, area under the curve; Clinical LDL C, clinical low‐density lipoprotein cholesterol; HC, healthy control; LA, linoleic acid; LDL C, low‐density lipoprotein cholesterol; LDL CE, cholesteryl esters low‐density lipoprotein; LDL FC, free cholesterol in low‐density lipoprotein; LDL L, total lipids in low‐density lipoprotein; LDL PL, phospholipids in low‐density lipoprotein; LEU, leucine; ML, machine learning; NON HDL C, total cholesterol minus high‐density lipoprotein cholesterol; OMEGA 6, Omega‐6 fatty acids; sMCI, stable mild cognitive impairment; TOTAL BCAA, total concentration of branched‐chain amino acids; TOTAL C, total cholesterol; TOTAL CE, total esterified cholesterol; TOTAL FC, total free cholesterol; TOTAL L, total lipids in lipoprotein particles; TOTAL PL, total phospholipids in lipoprotein particles; VAL, valine.

^a^ One‐way analysis of variance and Bonferroni post hoc comparisons.

^b^ Nonparametric statistics to independent samples with Kruskal–Wallis post hoc comparisons.

^c^ Multivariate analysis of covariance using age, sex, education, ethnicity, and race as covariates. Partial eta squared (pη^2^) value was greatest for: TOTAL BCAA (*pη* ^2 ^= 0.09) in the amino acids, TOTAL CE (*pη* ^2 ^= 0.19) in the cholesterol, OMEGA 6 (*pη* ^2 ^= 0.18) in the fatty acids, TOTAL PL (*pη* ^2 ^= 0.24) in the phospholipids, and TOTAL L (*pη* ^2 ^= 0.19) in the total lipids metabolite group.

### 3.2 Machine learning results

For all three binary classifications performed in the testing dataset, the ML models reached a specificity higher than 60% and a sensitivity higher than 50%. The AUC was different among the three models, reaching 0.89 and 0.68 for the classification of AD from HC and sMCI, respectively, and 0.60 for the classification of sMCI from HC. The AUC values of ML models (0.89, 0.60) were greater or similar to the ones obtained from Aβ (0.86, 0.59) and p‐tau (0.79, 0.59) values for AD and sMCI classifications from HC. The diagnostic performance of the ML model (0.68) was lower than that of Aβ (0.77) and p‐tau (0.72) for the classification of AD from sMCI.

The ReliefF test used to predict the target with fewer variables showed that 18, 24, and 22 variables were the most important features for distinguishing sMCI from HC, AD from HC, and AD from sMCI, respectively. Table 3 shows all the performance indices in the testing dataset for all the binary classifications when all variables were used and when only the most discriminative features were used in the models.

**TABLE 3:** ML performance indices for the test phases using all features (82 analytes, age, sex, education, and *APOE* ε4), and the most important predictors for each model. ROC analyses on Aβ42 and p‐tau181.

	sMCI (*n* = 250) vs HC (*n* = 250)		AD (*n* = 250) vs HC (*n* = 250)		AD (*n* = 250) vs sMCI (*n* = 250)	
Performance indices	All features	18 Predictors	All features	24 Predictors	All features	22 Predictors
ACCURACY	0.60	0.58	0.85	0.80	0.69	0.61
PPV	0.62	0.59	0.83	0.83	0.74	0.61
NPV	0.59	0.57	0.88	0.78	0.66	0.62
SENSITIVITY	0.52	0.52	0.89	0.76	0.61	0.59
SPECIFICITY	0.68	0.64	0.81	0.84	0.78	0.63
AUC	0.60	0.58	0.89	0.86	0.68	0.65

	sMCI (*n* = 198) vs HC (*n* = 122)		AD (*n* = 139) vs HC (*n* = 122)		AD (*n* = 139) vs sMCI (*n* = 198)	
	Aβ42	p‐tau181	Aβ42	p‐tau181	Aβ42	p‐tau181
Youden's Index	0.202	0.178	0.619	0.498	0.456	0.371
CUT POINT	847	17.4	820	23.1	997	23.7
SENSITIVITY	0.37	0.73	0.78	0.78	0.91	0.77
SPECIFICITY	0.83	0.45	0.84	0.72	0.55	0.60
AUC	0.59	0.59	0.86	0.79	0.77	0.72

Abbreviations: Aβ42, amyloid‐β42; AD, Alzheimer's disease; APOE ε4, Apolipoprotein E ε4 genotype; AUC, area under the curve; HC, healthy control; ML, machine learning; NPV, negative predictive value; PPV, positive predictive value; p‐tau181, phosphorylated tau181; ROC, receiver operating characteristic; sMCI, stable mild cognitive impairment.

### 3.3 Diagnostic features

Across the three binary classifications, *APOE* ε4, education, leucine, the total concentration of BCAAs, valine, the average diameter of low‐density lipoprotein (LDL) particles, and the ratio of omega‐6 to omega‐3 fatty acids, included in the amino acids, lipoprotein, and fatty acids categories, were the most predictive features. Sex, docosahexaenoic acid (fatty acids category), and glycerol (glycolysis‐related metabolite) were common predictors for the classification of sMCI and AD patients from HC. Alanine (amino acids category), sphingomyelins (other lipids), and glucose (glycolysis‐related metabolite) were the most important analytes for the classification of sMCI from HC and AD. Age, glyca (glycolysis‐related metabolite), histidine, phenylalanine (amino acids), and creatinine (fluid balance) were the most important variables for the classification of AD from HC and sMCI. Within the cholesterol category, only free cholesterol and cholesteryl esters in high‐density lipoprotein (HDL) effectively distinguished AD from HC and sMCI, respectively. In total, 39 of the 86 variables were selected as predictors across the models.

**FIGURE 2:**
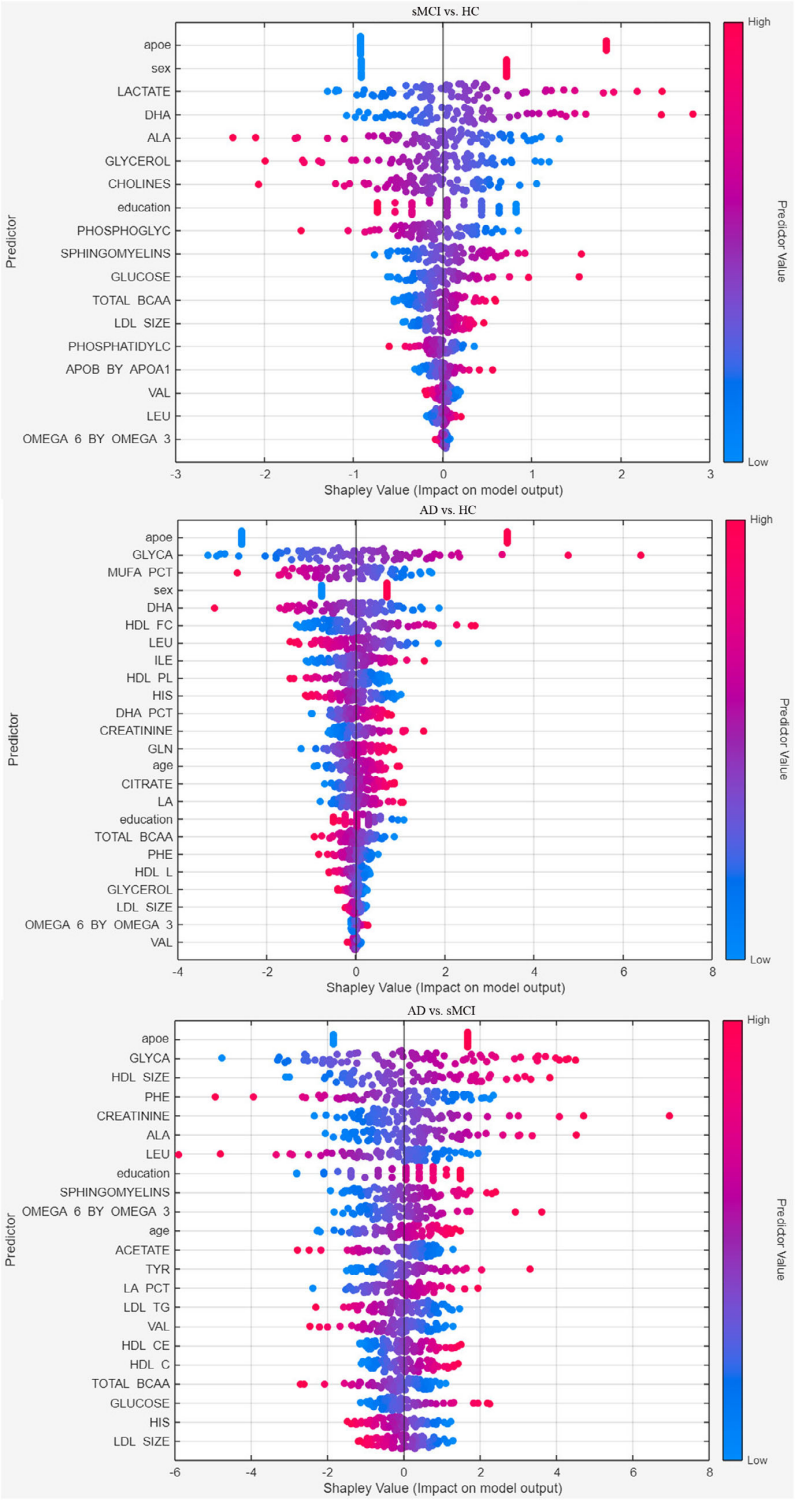
Shapley values distribution in the test dataset (*n* = 75) for the top predictors in the classification of sMCI from HC, of AD from HC, and AD from sMCI. Low and high values are shown in blue and red, respectively. AD, Alzheimer's disease; HC, healthy control; sMCI, stable mild cognitive impairment.

Figure 2 shows the Shapley values for the most important predictors for each binary classification model.

## 4 DISCUSSION

In this study, the statistical comparisons on the 82 analyzed circulating biomarkers revealed significant differences between AD and HC for 19 analytes belonging to seven metabolite categories. Among all significant analytes, the total phospholipids in lipoprotein particles showed the greatest statistical effect size (*pη* ^2 ^= 0.24). However, in the MANCOVA, for each of the seven significant metabolite groups, at least one analyte showed a medium or large effect size, suggesting that the combination of these analytes is clinically meaningful despite each analyte showing overlapping values among groups (Figure 1).

Leveraging a large sample size (750 subjects) and a high‐dimensional dataset (82 analytes, age, sex, education, and *APOE* ε4), we applied ML approaches to explore potential classification patterns. With the development of ML algorithms, there is growing interest in non‐invasive biomarkers obtained from serological tests, which could provide a potential approach for identifying systemic manifestations of cognitive deterioration and AD pathology.^3^, ^21^ Among the 82 possible blood‐based biomarkers used for creating the classification models, only a few analytes, mainly from amino acids, fatty acids, glycolysis‐related metabolites, lipoproteins, and other lipid categories, emerged as the most discriminative features for the ML‐based classification. Specifically, glycerol and docosahexaenoic acid in the fatty acids group ranked among the top predictors for classifying sMCI and AD versus HC. However, the model showed lower performance in classifying sMCI versus HC than in classifying AD versus HC. This result was in line with the poor ability to classify sMCI from HC by means of Aβ and p‐tau, and with the lack of significant differences between analyte values of sMCI and HC. The presence of *APOE* ε4 was the top predictor for all classification models, due to the significant higher prevalence in AD (63%) versus sMCI (44%) and HC (23%). A similar effect was observed for sex, which ranked among the most important predictors for distinguishing sMCI and AD from HC. Male sex was more prevalent in classifications toward sMCI (63%) and AD (59%) compared with HC (46%) (Figure 2).

**FIGURE 3:**
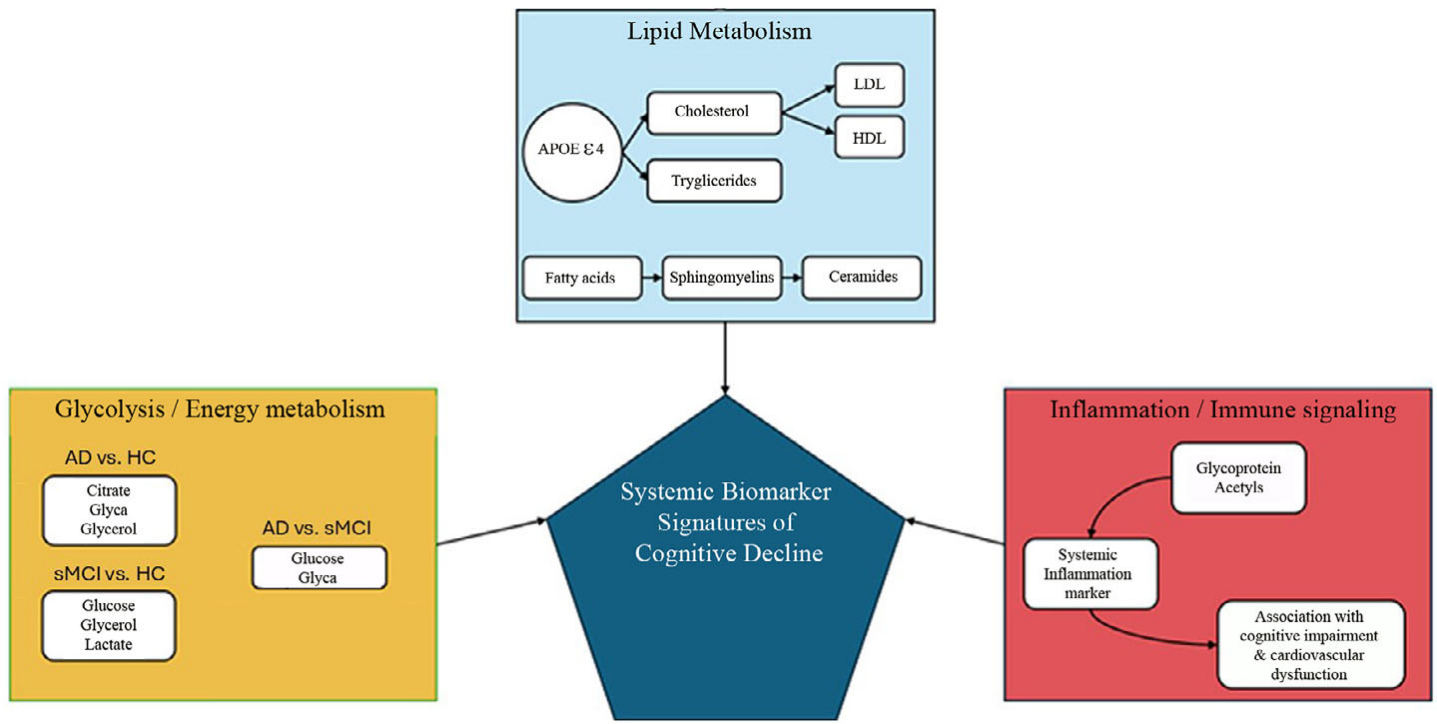
Biological pathways and peripheral biomarkers associated with cognitive decline across the AD continuum. The pictogram summarizes the systemic interactions between lipid metabolism, glycolysis/energy metabolism, and inflammation/immune signaling, highlighting key blood‐based biomarkers identified in the study. Lipid metabolism is strongly modulated by *APOE* ε4 status and includes alterations in cholesterol, triglycerides, fatty acids, and sphingomyelins, which may be converted into neurotoxic ceramides. Glycolysis‐related markers suggest metabolic dysregulation with relevance to mitochondrial dysfunction and the “type 3 diabetes” hypothesis of AD. Inflammation is indexed by glycoprotein acetyls, a systemic marker associated with cognitive impairment and cardiovascular dysfunction. Together these biomarkers represent systemic signatures predictive of the transition from healthy control (HC) to mild cognitive impairment (MCI) and Alzheimer's disease (AD), as identified by machine learning models. APOE, apolipoprotein E; HDL, high‐density lipoprotein; LDL, low‐density lipoprotein; sMCI, stable mild cognitive impairment.

Previous studies reported associations between the apoE ε4 variant and plasma levels of fatty acids,^22^ blood triglyceride,^23^ and cholesterol concentrations^24^ due to the interaction of fatty acids, lipid metabolism, and *APOE*.^25^ Other studies showed that elderly individuals with elevated plasma cholesterol have an increased susceptibility to dementia and AD^26^ compared with normocholesterolemic subjects, an effect likely mediated by defective cholesterol catabolism in *APOE* ε4 carriers.^27^, ^28^ Although brain cholesterol is exclusively synthesized in situ, peripheral cholesterol alterations may act as a proxy of central disturbances, especially in *APOE* ε4 carriers. Our study showed altered cholesterol analytes in patients with AD (Table 2), confirming the possible association with *APOE* ε4 status and cholesterol alterations.^29^ Lipids and triglycerides, which were involved in ML models, are closely associated with cognitive deficits in AD.^29^ Besides their role as energy substrates, triglycerides can participate in neuroinflammatory processes mediated by astrocyte and microglia activation.^30^ The idea that some of the reported alterations have a neuroinflammatory drive is further supported by changes in glycoprotein acetyl levels, an inflammatory marker. Indeed, glyca was the most important peripheral biomarker for distinguishing AD from HC and sMCI individuals (Figure 2). Altered levels of glycoprotein acetyls have been associated with poorer cognitive performances,^31^ and they have recently emerged as novel biomarkers of inflammation‐related cardiovascular dysfunction,^32^ pointing to a possible mechanistic link between chronic systemic inflammation and cerebrovascular contributions to neurodegeneration (Figure 3).

Glycolysis‐related metabolites were critical features for distinguishing sMCI from HC (i.e., lactate, glycerol, and glucose) and AD (glyca and glucose) (Figure 3). Metabolites related to glycolysis are associated with type 2 diabetes, and their dysregulation has been strongly linked to memory deficits, cognitive decline, and many of the characteristic symptoms that have been displayed in AD.^15^ Previous studies have suggested that AD may represent a brain‐specific form of diabetes, often referred as a “type 3” form of diabetes.^33^, ^34^ Although impaired insulin signaling in AD is distinct from that in diabetes mellitus, the peripheral glycolysis‐related alterations identified in our analysis may represent an early warning sign of broader systemic endocrine changes that reflect underlying neuroendocrine disturbances. Whether this role of glucose translates into pathological cognitive decline or increases dementia risk in old age remains unclear.^35^ Taken together, these findings suggest that type 2 diabetes acts as a cofactor in the pathogenesis and progression of AD, and, at the same time, might be reflective of a much broader endocrine alteration that also affects the brain.^33^ Overall, our findings reinforce the hypothesis that metabolic dysregulation contributes to both the onset and progression of AD, possibly serving as a systemic marker of neurodegenerative processes.^36^ Previous studies showed that, in preclinical mouse models of AD, the astrocyte‐neuron lactate shuttle is required for long‐term memory formation,^37^ a process found to be impaired in AD settings.^38^ Neuroprotective properties are also associated with fatty acids in cognitive aging studies.^39^

It is important to notice that in our study design, we included only sMCI patients who did not convert to dementia within 36 months after blood collection. The sMCI group also had a lower prevalence of *APOE* ε4 carriers (44%) compared to the AD group (63%). This may suggest that the sMCI cohort represents individuals at a preclinical disease stage or with alternative pathological pathways distinct from AD dementia.

Our ML classification models also showed that leucine, valine, the BCAA, the average diameter for LDL particles, and the ratio of omega‐6 to omega‐3 fatty acids included in amino acids and lipoprotein and fatty acids categories were the most predictive features for all three classification models (Figure 2). Alanine from the amino acids category and sphingomyelins were common predictors for distinguishing sMCI from HC and AD. The link between BCAAs and the risk of AD is complex and poorly understood, with conflicting findings suggesting that these metabolites may not be a direct causal factor.^40^, ^41^ Conversely, elevated levels of sphingomyelin were consistently found in the brain and in the CSF of AD patients compared to non‐demented controls.^42^, ^43^, ^44^, ^45^ A potential mechanistic link involves the conversion of sphingomyelin into ceramides, bioactive lipids (not present in our dataset) known to contribute to neuronal aging, cell death, and the progression of AD.^46^, ^47^ Overall, these findings are consistent with previous studies suggesting that amino acids, lipids, immune, inflammatory, and metabolic biomarkers—along with their complex, non‐linear interactions—collectively represent a biological signature of cognitive health.^48^, ^49^

In conclusion, the findings obtained from traditional statistical analysis and ML indicate that the subset of analytes highlighted by both approaches can effectively separate individuals with MCI or AD from HC. Although not all analytes are part of routine clinical chemistry panels, our findings support the utility of accessible metabolomic profiling, which is becoming increasingly feasible in specialized clinical and translational research settings. Our ML models help to identify a subset of blood‐based biomarkers for the classification of sMCI from HC and AD and for AD from HC and sMCI (Figure 3).

However, several challenges remain in widespread ML applications, including data heterogeneity, model interpretability, and limited generalizability.

In our study, the sample size of each group was balanced in the training, validation, and test phases. Indeed, the imbalance of groups can lead to a falsely perceived positive effect of a model's accuracy, because the input data has bias toward one class, which results in the trained model mimicking that bias. In addition, potential overfitting of the ML algorithms was reduced using unseen data as a test dataset and performing the hyperparameter optimization.

In our study model, explainability was enhanced using the SHAP technique. However, collaborative efforts between technologists and clinicians are essential to unlock the full potential of ML in Alzheimer's research and care. In addition, a crucial step toward the clinical implementation of accurate prediction models is external validation, which is necessary to determine a prediction model's reproducibility and generalizability to different cohorts of patients across various regions, countries, or care settings.

The utilization of blood‐based biomarkers offers a less‐invasive, more accessible, and cost‐effective alternative to traditional diagnostic methods such as CSF analysis, and MRI and PET scans.

Future research should integrate data on Aβ deposition, tau accumulation, and NfL levels with blood‐based biomarkers and should stratify or adjust for key confounders such as body mass index, comorbidities (e.g., diabetes, cardiovascular disease), and medications. Such comprehensive analyses could enhance our understanding of the relationship between peripheral biomarker alterations and AD pathology, potentially leading to more accurate and early diagnoses (Supporting Information).

## CONFLICT OF INTEREST STATEMENT

The authors have no conflicts of interest to disclose. Any author disclosures are available in the supporting information.

## CONSENT STATEMENT

The Alzheimer's Disease Neuroimaging Initiative (ADNI) project received approval from the institutional review boards of all participating institutions, and all participants provided informed written consent.

## Supporting information



Supporting Information

## Data Availability

The original data used in this manuscript are available for download in the ADNI database (https://adni.loni.usc.edu).
